# Effect of a novel dietary supplement Khejri, and Spirulina supplementation on lipid profile in cricket players

**DOI:** 10.3389/fspor.2022.1075388

**Published:** 2023-01-16

**Authors:** Arvind Pareek, Bhanwra Ram Kasvan, Neha Singh

**Affiliations:** ^1^ Department of Botany, Maharshi Dayanand Saraswati University, Ajmer, India; ^2^ Department of Sports Bioscience, Central University of Rajasthan, Ajmer, India

**Keywords:** supplements, cholesterol, Spirulina, performance, lipid profile, cricket players

## Abstract

*Prosopis cineraria* (Fabaceae) is known as Khejri in India or the golden tree of Indian deserts. It’s potential as a dietary supplement in sports nutrition and its effect on regulating lipid profile has never been investigated. Spirulina (*Arthrospira platensis*) is a superfood with high nutritional value and is a popular supplement among athletes. In the current study, Spirulina and Khejri were used as supplements by cricket players to improve their physical fitness and lipid profile. Both supplements were given to individual groups and in combination to see the combined effect. The intervention period was 21 days, and supplements were given in 500 mg doses daily. Lipid profile assessments were done before and after the intervention period. 40 cricket players were divided into 4 groups: Group 1 (*n* = 10): Both supplements, Spirulina and Khejri, Group 2 (*n* = 10): Supplement Spirulina, Group 3 (*n* = 10): Supplement Khejri, and Group 4 (*n* = 10): Control. When experimental groups 1, 2 and 3 were compared to the control group 4, significant reduction was observed in triglyceride levels (Group1 vs. control: 141.53 ± 14.74 vs. 199.28 ± 27.24, *p* < 0.05; Group 2 vs. control: 137.5 ± 14 vs. 199.28 ± 27.24, *p* < 0.05; Group 3 vs. control: 135.32 ± 17.34 vs. 199.28 ± 27.24, *p* < 0.05) and significant reduction in cholesterol levels was found post-intervention after 21 days of supplementation (Group1 vs. control: 149.75 ± 7.08 vs. 207.86 ± 11.69, *p* < 0.001; Group 2 vs. control: 178.28 ± 9.43 vs. 207.86 ± 11.69, *p* < 0.05; Group 3 vs. control: 142.92 ± 10.01 vs. 207.86 ± 11.69, *p* < 0.001). Cholesterol and Triglyceride levels were significantly decreased pre- vs. post-intervention by Khejri and Spirulina supplements in cricket players.

## Introduction

Cricket is an international sport, and cricket players maintain their physical fitness by following specific exercise training programs to meet the game's physiological demands and other performance requirements (1). Differences may exist in particular nutrient needs along this designated spectrum of cricketers, creating the exciting challenge of individualizing sports nutrition plans. The quality and selection of food are an important part of a diet for a cricket player. Players need to plan their carbohydrate, protein, and vitamin intake (2, 3). Physical fitness and optimal nutrition, including dietary supplements as per requirement, are of immense importance for sports person and are an important and essential aspect of achieving optimum health and performance for athletes (2, 4, 5). The nutritional requirements of cricket are specific to the game's longer duration and physiological demands. A balanced diet with adequate energy, macro and micronutrients is the primary requirement for the cricket player (6). For optimal performance, cricket players require sufficient carbohydrates for energy during intense training and protein for repairing and adaptation to training (7). Along with whole foods and drinks, dietary supplements like creatine monohydrate, caffeine, beta-alanine, sodium bicarbonate, omega 3 fatty acids, probiotics, etc., help in enhancement of general muscle and brain performance, in addition, boosting adaptations to exercise and recovery in athletes (8).

## Herbal supplements in sports

Herbal supplements are becoming popular among athletes because of their rich nutritional profile and performance-enhancing potential (9). Athletes prefer herbal supplements for enhancing exercise performance, promoting muscle growth, inducing weight loss, increasing energy, and increasing mental alertness (10). Several herbal supplements are popular among athletes; some are listed here: Ginseng, Caffeine, Ginkgo biloba, Saffron, etc. (10). These supplements have nutritional benefits for sportspersons, although the research on these herbal supplements is also minimal. Therefore, this study was focused on identifying and validating novel herbal dietary supplements which can help cricket players to improve their lipid profile, cardiovascular health, and fitness. Khejri alone and Spirulina and Khejri in combination have never been used earlier as a dietary supplement by cricket players, which defines the utmost importance and future applications of observations of this study.

## Spirulina as supplement in sports

Spirulina (*Arthrospira platensis*) is a popular supplement that is known for its high nutritional value. Ancient Aztecs used it as a protein-rich supplement in their diets. The recorded history of the consumption of microalgae dates back to the year 1521 when Bernal Diaz del Castillo described the consumption of a preparation of Spirulina by people in the vicinity of Mexico City. Due to its unmatched composition, it was declared as “best food for tomorrow” by United Nations at the world food conference in 1974 (11, 12). Spirulina contains all the amino acids required by the human body. In addition, it includes vitamins, antioxidants, and other vital nutrients. Moreover, it also improves gut health and thus boosting energy production resulting in improved sports performance (13). Furthermore, it has multiple bioactive compounds and is also known to improve brain health (14). It is a primary source of protein, with high nutritive value and multiple health benefits. The energetic value of Spirulina comes from its ingredients, including fat, protein, carbohydrate, vitamin A, vitamin B1 (thiamine), B2 (riboflavin), B3 (nicotinamide), B6 (pyridoxine), B9 (folic acid), vitamin C, vitamin E, natrium, potassium, calcium, magnesium, iron and other minerals (15). It is rich in antioxidants and has fatigue-delaying capabilities. Therefore, being a functional food rich in nutrients, it is known to boost exercise performance and has huge ergogenic potential in sports nutrition (16).

## Khejri as supplement in sports

*Prosopis cineraria* are commonly known as Khejri. It is a leguminous multipurpose tree; its bark is helpful in the treatment of cough, common cold, asthma, leukoderma, piles, and tremors of the muscles (17). Khejri has socio-economic importance for farmers; it is used for fuel, firewood, and charcoal. Dry pods of Khejri are known as sangri and are the main part of Rajasthani dishes. Khejri also, has therapeutic and pharmaceutical value; it helps relieve pain, lowers cholesterol levels, and is used to treat diabetes, anemia, and liver disorders. Khejri leaves have high nutrient content and antibacterial, anti-hyperglycemic, and anti-oxidant activity (17). Flowers are used as a blood purifier and for skin treatment. The bark is used to treat cough, dysentery, asthma, files, and tremors of the muscles. Khejri has never been used as a supplement in sports nutrition to date. So, we considered the effects of Khejri and Spirulina supplements on cricket players’ fitness and lipid profile.

## Methods

In this research work, we conducted a comparative study of the effect of herbal supplementation on the physical fitness and lipid profile of cricket players. A total of 40 cricket players (Mean ±  SEM, age in years 23.53 ± 0.55) participated in this study and were divided into 4 groups. Khejri and Spirulina were used as supplements by cricket players to improve their physical fitness and body composition. Dried pods of Khejri are known as Sangri and are the main part of Rajasthani cuisine. Both supplements were given to individual groups and in combination to see the combined effect of both supplements. The intervention period was 21 days, and supplements were given in 500 mg doses daily. Lipid profile assessments were done before and after the intervention period. 40 cricket players were divided in 4 groups: Group 1 (*n* = 10): Both supplements Spirulina and Khejri B, Group 2 (*n* = 10): Supplement Spirulina, Group 3 (*n* = 10): Supplement Khejri, and Group 4 (*n* = 10): Control. The inclusion criteria included healthy young, physically active cricket players involved in this study. The exclusion criteria included individuals with a cardiorespiratory disease or on medication. After using inclusion or exclusion criteria, overall, 40 samples were collected. A professional lab assistant collected blood. Blood samples (5 ml) for determining lipid profiles were obtained at rest. The blood was centrifuged at 3,000 rpm for 5 min at 4°C for serum separation. All of the biochemical tests have been done using serum samples. Lipid parameters (Triglyceride; Cholesterol) were measured using Arkray kits and a Biochemistry analyzer (RMS).

Statistical analyses were performed using SPSS software. Shapiro Wilk test was used to test the normality of data. Descriptive statistics were expressed as mean (±SEM) for each variable. For the data collected in the current study, the mean value, standard deviation, standard error of mean, one way ANOVA and independent “*t*” test was applied to analyse the data. The level of significance for all tests was at *p* < 0.05.

### Ethical consideration

Cricket players were invited to participate in a physical fitness test. They provided a consent form to fill in their general information, consent to use their data in this research, and agreement for their volunteer participation. Ethical approval for the study was obtained from the Ethics Committee, and informed consent forms were obtained from all players.

## Results

**Figure 1:**
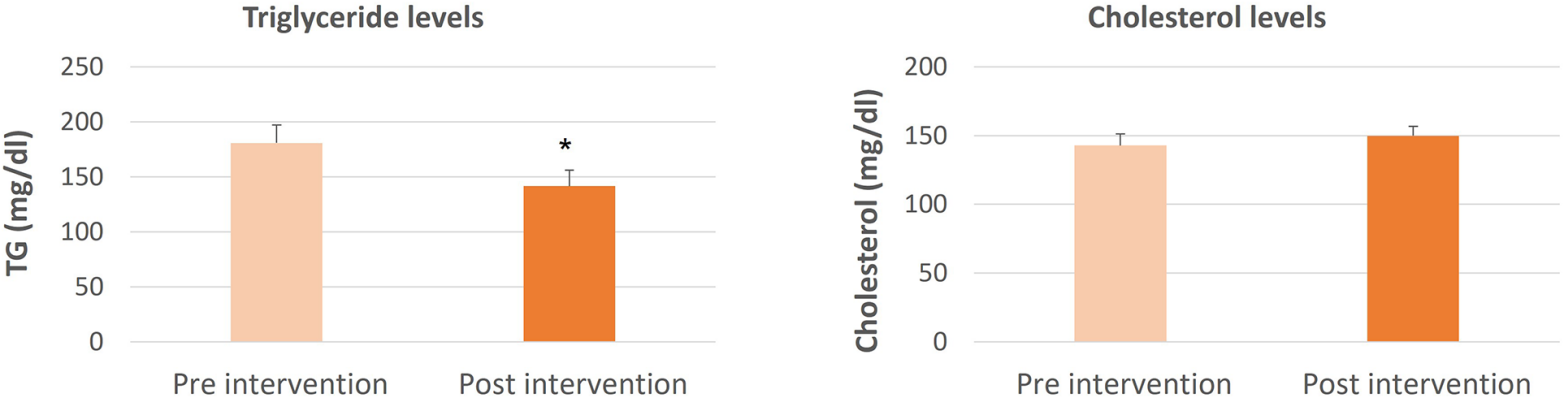
Triglyceride and cholesterol levels comparison in cricket players (*n* = 10) between pre and post-intervention period of 21 days supplementation with Spirulina and Khejri in combination, *p < 0.05, **p < 0.01, ***p < 0.001.

**Figure 2:**
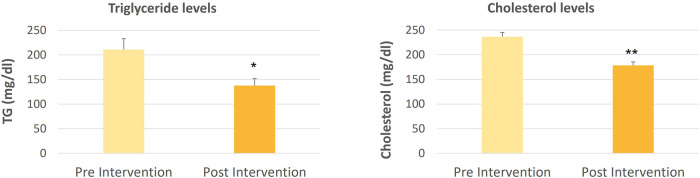
Triglyceride and cholesterol levels comparison in cricket players (*n* = 10) between pre and post-intervention period of 21 days supplementation with Spirulina,*p < 0.05, **p < 0.01, ***p < 0.001.

**Figure 3:**
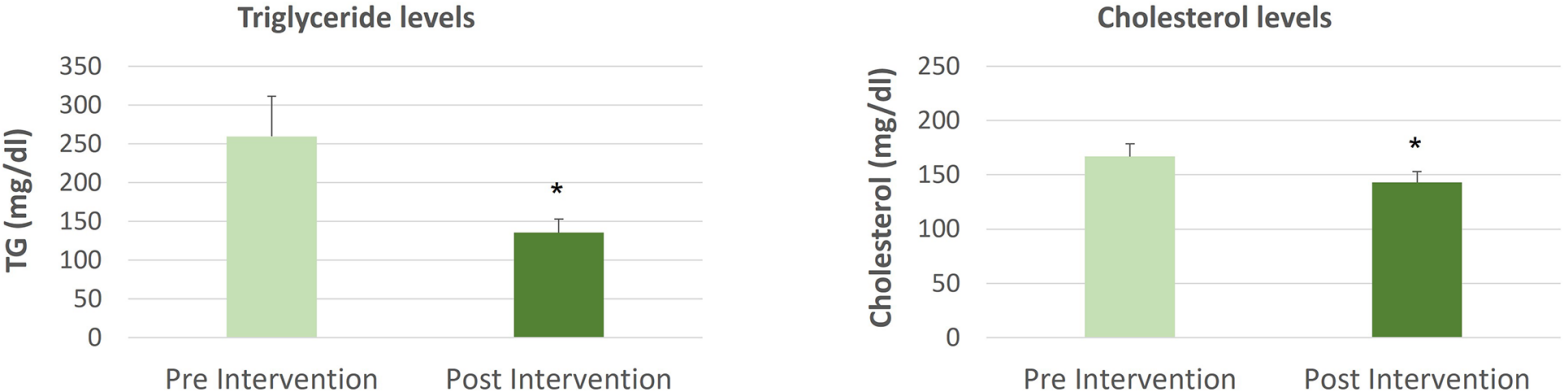
Triglyceride and cholesterol levels comparison in cricket players (*n* = 10) between pre and post-intervention period of 21 days supplementation with Khejri, *p < 0.05, **p < 0.01, ***p < 0.001.

**Figure 4:**
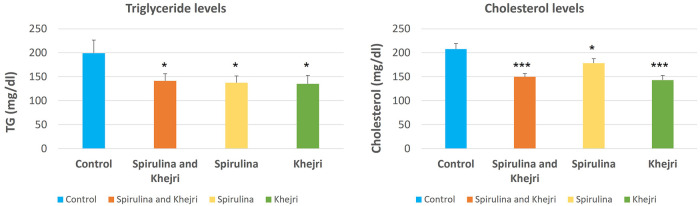
Triglyceride and cholesterol levels comparison in cricket players between all the 4 groups: Group 1 (*n* = 10): both supplements Spirulina and Khejri B, Group 2 (*n* = 10): supplement Spirulina, Group 3 (*n* = 10): supplement Khejri, Group 4 (*n* = 10): control, *p < 0.05, **p < 0.01, ***p < 0.001.

The data for comparing cholesterol and triglyceride levels pre and post-intervention with Spirulina and Khejri supplementation in cricket players are shown in Figures 1–4. The one-way ANOVA for comparison between the treated groups yielded a statistically significant effect for cholesterol levels, *p* < 0.05. However, for triglyceride levels, the one-way ANOVA for comparison between treated groups was not found to be significant, *p* = 0.06. The results illustrate that the triglyceride level is significantly lowered post-intervention in Group 1 with Spirulina and Khejri combination supplementation (180.88 ± 16.45 vs. 141.53 ± 14.74, *p* < 0.05). However, no significant difference was observed in cholesterol levels post-intervention in the same group (142.87 ± 8.4 vs. 149.75 ± 7.08, *p* > 0.05) (Figure 1). In Group 2, supplementation with Spirulina alone was found to lower triglyceride levels significantly in cricket players (210.82 ± 22.28 vs. 137.5 ± 14, *p* < 0.05) as well as cholesterol levels were reduced significantly post-intervention (236.47 ± 10.44 vs. 178.28 ± 9.43, *p* < 0.01) (Figure 2). In Group 3, supplementation with the novel sports nutrition supplement Khejri resulted in significantly lower triglyceride levels (259.51 ± 51.76 vs. 135.32 ± 17.34, *p* < 0.05) and cholesterol levels (166.91 ± 11.64 vs. 142.92 ± 10.01, *p* < 0.05) post-intervention in cricket players (Figure 3). When all the three experimental groups 1, 2 and 3 were compared to the control group 4, significant reduction was observed in triglyceride levels (Group 1 vs. control: 141.53 ± 14.74 vs. 199.28 ± 27.24, *p* < 0.05; Group 2 vs. control: 137.5 ± 14 vs. 199.28 ± 27.24, *p* < 0.05; Group 3 vs. control: 135.32 ± 17.34 vs. 199.28 ± 27.24, *p* < 0.05) as well as significant reduction in cholesterol levels was found post-intervention after 21 days of supplementation (Group 1 vs. control: 149.75 ± 7.08 vs. 207.86 ± 11.69, *p* < 0.001; Group 2 vs. control: 178.28 ± 9.43 vs. 207.86 ± 11.69, *p* < 0.05; Group 3 vs. control: 142.92 ± 10.01 vs. 207.86 ± 11.69, *p* < 0.001) (Figure 4).

## Discussion

This study aimed to study the effect of novel dietary supplement Khejri and Spirulina on cricket players’ lipid profiles. After the supplementation intervention period of 21 days, cholesterol and triglyceride levels reduced significantly compared to the control group and to pre-intervention levels for each Khejri and Spirulina supplementation when taken in combination. The observation that triglyceride and cholesterol levels decreased with Spirulina supplementation is in agreement with previous studies (18, 19). However, such analysis has never been done on cricket players. In addition, investigating the effect of the combination of Spirulina and Khejri supplementation and Khejri supplementation alone to improve lipid profile and fitness is the first-ever study done on cricket players. Weight loss is one of the common goals for athletes in any kind of sports (20). Cricket players also need to maintain their weight and fitness. It is a highly novel study with high-impact findings in sports nutrition. This suggests that it is valuable information for cricket players who can maintain their weight and lipid profile without taking synthetic or harmful supplements. To achieve optimal sports performance, cricket players exercise regularly, undergo training, take a balanced diet, and choose healthy fats and a healthy lifestyle. To achieve and maintain such fitness, many cricket players tend to consume synthetic supplements that can have very harmful side effects (21, 22). Considering multiple benefits and negligible side effects of herbal dietary supplements, supplementation with novel plant-based nutraceuticals has become a common practice among athletes. Herbal supplements are an alternative to avoid harmful side effects (10). Spirulina has also been shown to work synergistically with exercise in improving cardiorespiratory fitness, body composition, and blood lipid profile (23).

Since triglyceride and cholesterol levels are an essential measure of cardiovascular health (24), it is important for cricket players to maintain their cardiovascular health by including natural supplements in their diet, such as Spirulina and Khejri. Several studies have demonstrated the ergogenic potential of Spirulina in sports performance. A study by Johnson et al. showed improvement in physical and mental fatigue in men after spirulina supplementation (25). Another study by Zeinalian et al. demonstrated the efficacy of Spirulina in the control of obesity (26). The hypolipidaemic effects of spirulina supplementation have been shown in multiple studies (27–29). Khejri is an entirely new dietary supplement that has not been studied for its benefits on sports performance. Its effect on lipid profile was also not studied until now. With this study, we have tried to add valuable information to scientific literature about the lipid profile improvement benefits of Khejri and for further research on its action mechanism and other possible potential benefits not only for cricket players but also, for athletes in other sports.

In conclusion, our data suggest that the dietary supplements Khejri and Spirulina confer a beneficial effect on the cardiovascular and overall health of cricket players. Appropriate nutrition is very important, along with regular exercise and training for the fitness of cricket players and better sports performance.

## Novelty

The study's novelty is elaborated in the following points: (1) Khejri is a novel supplement that was never used earlier as a dietary supplement in sports nutrition. (2) Combination of Khejri and Spirulina is also a new addition to sports nutrition. (3) This is the first-ever study to report such intervention and its benefits for cricket players’ fitness and cardiovascular health.

## Practical application

Cricket is one of the most popular sports in India. Identifying and establishing the health benefits and ergogenic efficacy of such herbal supplements for cricket players is extremely important. It can also be developed into a new herbal supplement with no harmful side effects.

## Data Availability

The original contributions presented in the study are included in the article/**Supplementary Material**, further inquiries can be directed to the corresponding author.
