# Supplementary figures and images for: Effect of a novel dietary supplement Khejri, and Spirulina supplementation on lipid profile in cricket players
Source: Front Sports Act Living. 2023 Jan 16;4:1075388. doi: 10.3389/fspor.2022.1075388 (PMC9885189; doi:10.3389/fspor.2022.1075388)

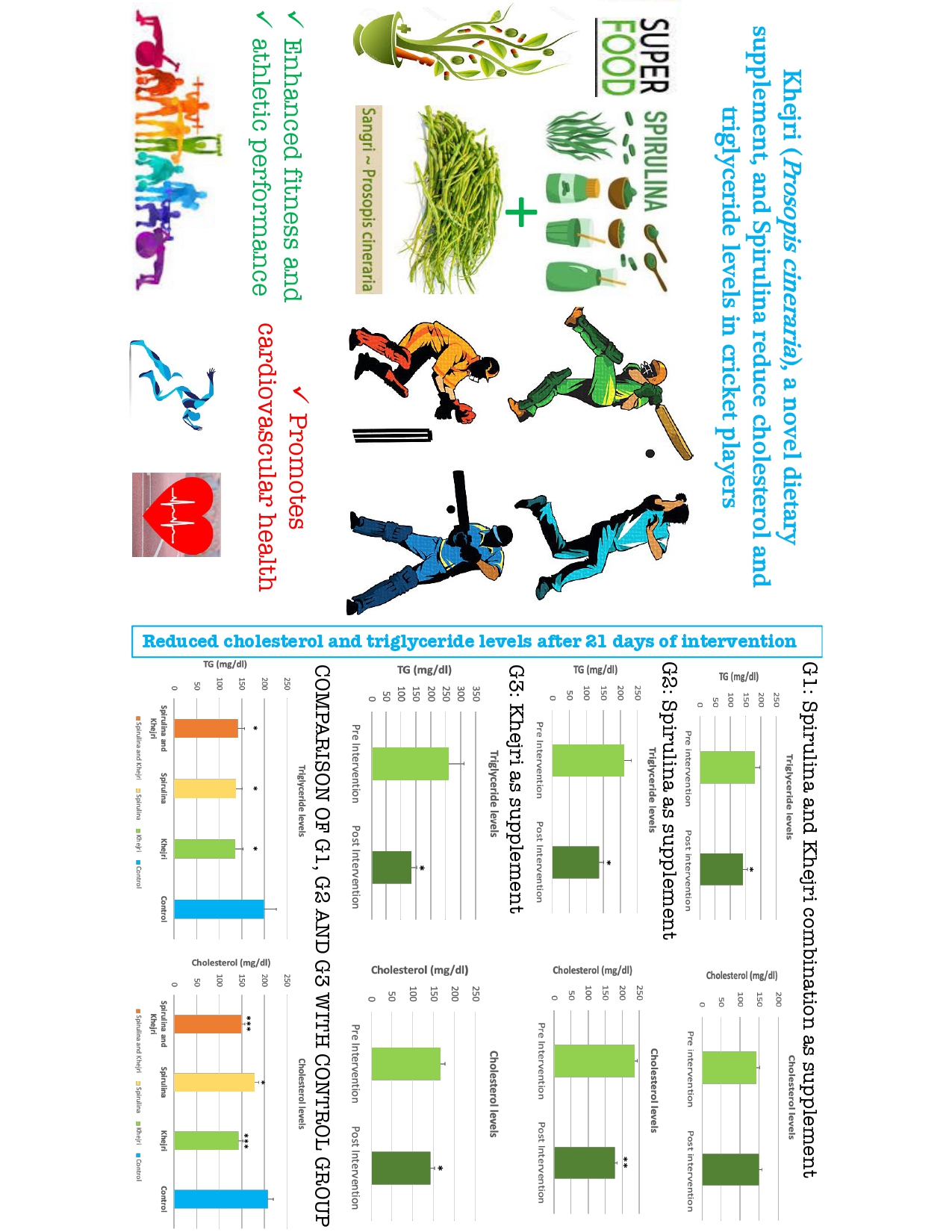

Supplement: Supplementary file 1 [file Image1.jpeg]
